# Adaptive Dual-Bias Estimation for Hybrid Inertial Navigation with Single-Axis Cold Atom Interferometric Gyroscope

**DOI:** 10.3390/s26206379

**Published:** 2026-10-09

**Authors:** Qiang Guo, Jianfei Zhou, Xuguang Yue, Jun Cheng, Chen Huang, Changhong Chen

**Affiliations:** 1 Wuhan National Laboratory for Optoelectronics, Huazhong University of Science and Technology, Wuhan 430074, China; guoqiang@cssc717.com; 2 Huazhong Institute of Electro-Optics, Wuhan 430223, China; zhoujianfei@cssc717.com (J.Z.); yuexuguang@cssc717.com (X.Y.); chengjun@cssc717.com (J.C.); huangchen@cssc717.com (C.H.); 3 School of Mechanical Science & Engineering, Huazhong University of Science and Technology, Wuhan 430074, China

**Keywords:** cold atom interferometric gyroscope, hybrid inertial navigation, gyroscope bias estimation, adaptive Kalman filter

## Abstract

The errors of an inertial navigation system accumulate over time due to its operating principles and sensor limitations. Numerous studies have investigated hybrid inertial navigation schemes combining a cold atom interferometric gyroscope (CAIG) and a conventional inertial measurement unit. This paper constructs a dual-bias joint state model to realize decoupling of gyroscope biases between the conventional gyroscope and the CAIG, eliminating modeling error introduced by the drift-free reference sensor assumption. Furthermore, an adaptive Kalman filtering algorithm based on real-time residual autocorrelation identification is proposed to cope with the temporally correlated time-varying noise characteristics. A series of simulations including noise-characteristic verification and bias-decoupling comparison, as well as static physical experiments, are carried out to validate the bias-decoupling capability and overall navigation performance. Taking advantage of the low bias-drift property of the CAIG and the high sampling rate of conventional gyroscopes, static experimental results show that the hybrid navigation system with a single-axis CAIG reduces positioning errors by more than 85% after compensating for inertial sensor biases, and achieves a positioning accuracy of 5 nautical miles over a one-hour test. Although the current validation is limited to stationary conditions, this work provides a feasible solution for CAIG-aided hybrid inertial navigation.

## 1. Introduction

An inertial navigation system (INS) utilizes acceleration and angular-rate measurements from inertial sensors to generate precise navigation solutions, including attitude, velocity, and position [1]. Benefiting from full autonomy, passive operation, and high real-time performance, INSs have been widely used in both civilian and military scenarios, covering land, marine, airborne, and space navigation applications [2]. As a dead-reckoning method, INSs inherently suffer from time-dependent error accumulation, which leads to gradual positioning divergence [3,4]. To suppress such cumulative errors, global navigation satellite systems (GNSSs) are commonly adopted to improve overall navigation accuracy [5]. Various filtering techniques, such as the Kalman filter and its variants, have been widely used in GNSS/INS integration to fuse the complementary measurements [5,6,7]. Nevertheless, satellite-aided navigation is vulnerable to complex environments. Urban canyons, tunnels, and underwater scenarios frequently block satellite signals, resulting in severe performance degradation of GNSS/INS integrated navigation under GNSS-denied conditions. Moreover, once satellite signals are completely lost, GNSS/INS hybrid navigation fails to maintain the required navigation accuracy [6,7].

Extensive research has been conducted to enhance pure INS performance, covering initial alignment optimization [8], damping design [9], and multi-INS collaboration navigation [10,11]. Nevertheless, further performance improvement in inertial navigation accuracy is fundamentally restricted by the limitations of inertial sensor hardware, especially gyroscope performance [12,13]. As a new-generation gyroscope technology, the cold atom interferometric gyroscope (CAIG) operates based on the Sagnac effect of the atomic de Broglie wave. Since the first demonstration of an atom interferometric gyroscope using stimulated Raman transitions in 1997 [14], CAIG technology has been extensively investigated by numerous research groups [15,16,17]. With advances in cold-atom technology, CAIG has been successfully applied in fundamental scientific fields such as geophysics and metrology [18], primarily for high-precision physical constant measurements, such as the Earth’s rotation constant [19]. As the gyroscope is the core rotation-sensing component of an inertial measurement unit (IMU), the CAIG with ultra-high-precision and long-term stability has attracted considerable research interest in inertial navigation [20,21,22]. Despite the significant theoretical advantages, practical navigation applications of the CAIG still face multiple technical constraints, such as limited dynamic range and a low output data rate [23].

Currently, fusing CAIGs as aiding sensors for conventional high-rate IMUs has become a promising research direction, which can simultaneously combine the high data rate of traditional gyroscopes and the long-term stability of the CAIG [24,25]. The existing CAIG-aided inertial navigation frameworks still exhibit obvious limitations. Generally, the CAIG is treated as a drift-free reference sensor, and only the errors of conventional gyroscopes are estimated [26,27]. Such simplified modeling strategies cause coupled errors between dual gyroscopes, thereby introducing systematic deviations that cannot be eliminated through traditional filtering frameworks. Furthermore, existing studies generally ignore the non-ideal time-varying characteristics of the CAIG, including slowly varying weak biases and non-stationary colored noise under long-duration operation [28,29].

To address the aforementioned problems, this paper proposes a dual-bias joint estimation model and an adaptive Kalman filtering algorithm. By combining the precision advantage of the CAIG and the high-update-rate characteristic of a conventional IMU, a hybrid inertial navigation scheme is constructed. Different from the traditional single-bias estimation strategies, the dual-bias state model simultaneously estimates the biases of a conventional gyroscope and the CAIG, which fundamentally realizes the decoupling of gyroscope biases and eliminates systematic modeling errors introduced by the drift-free assumption. Furthermore, an adaptive Kalman filtering algorithm based on real-time residual autocorrelation identification is designed, so as to improve the stability and accuracy of the hybrid navigation system. All simulations and physical experiments in this work are implemented based on a single-axis CAIG platform under static conditions.

The remainder of this paper is organized as follows. Section 2 presents the system framework of the hybrid navigation system, together with the dual-bias estimation model and the adaptive Kalman filtering algorithm. Section 3 details the simulation analysis. Section 4 presents the static experimental platform, test results and discussion. Finally, conclusions are given in Section 5.

## 2. System Model and Algorithm

### 2.1. Hybrid Inertial Navigation Framework

The cold atom interferometer gyroscope (CAIG) measures rotational inertial angular velocity based on a three-pulse cold atom interferometry configuration. The operational procedure of the CAIG is mainly divided into atom cloud preparation, cooling, state selection, the interference process, and final atomic state detection.

Specifically, Rb87 atoms are trapped in a three-dimensional magneto-optical trap (3D-MOT), and then launched along the positive x-axis. After that, atoms are split, reversed, and recombined by three pairs of Raman pulses separated by a fixed time interval T, which generates interference. The interference phase shift primarily originates from the phase of the Raman laser acting on the atoms. By measuring the population of atomic energy states after interference, phase information can be extracted to calculate inertial angular velocity. The trapping and manipulation of the atom cloud are all realized by laser control technology, which requires a precise optical system for laser frequency and phase. In order to achieve better measurement accuracy and reduce the influence of the external environment, the atomic interference process needs stable vacuum, temperature, magnetic shielding, and vibration isolation. Here we only briefly summarize the most important and fundamental principles of the three-pulse CAIG. For further details and more rigorous descriptions, readers are referred to [30,31].

**Figure 1:**
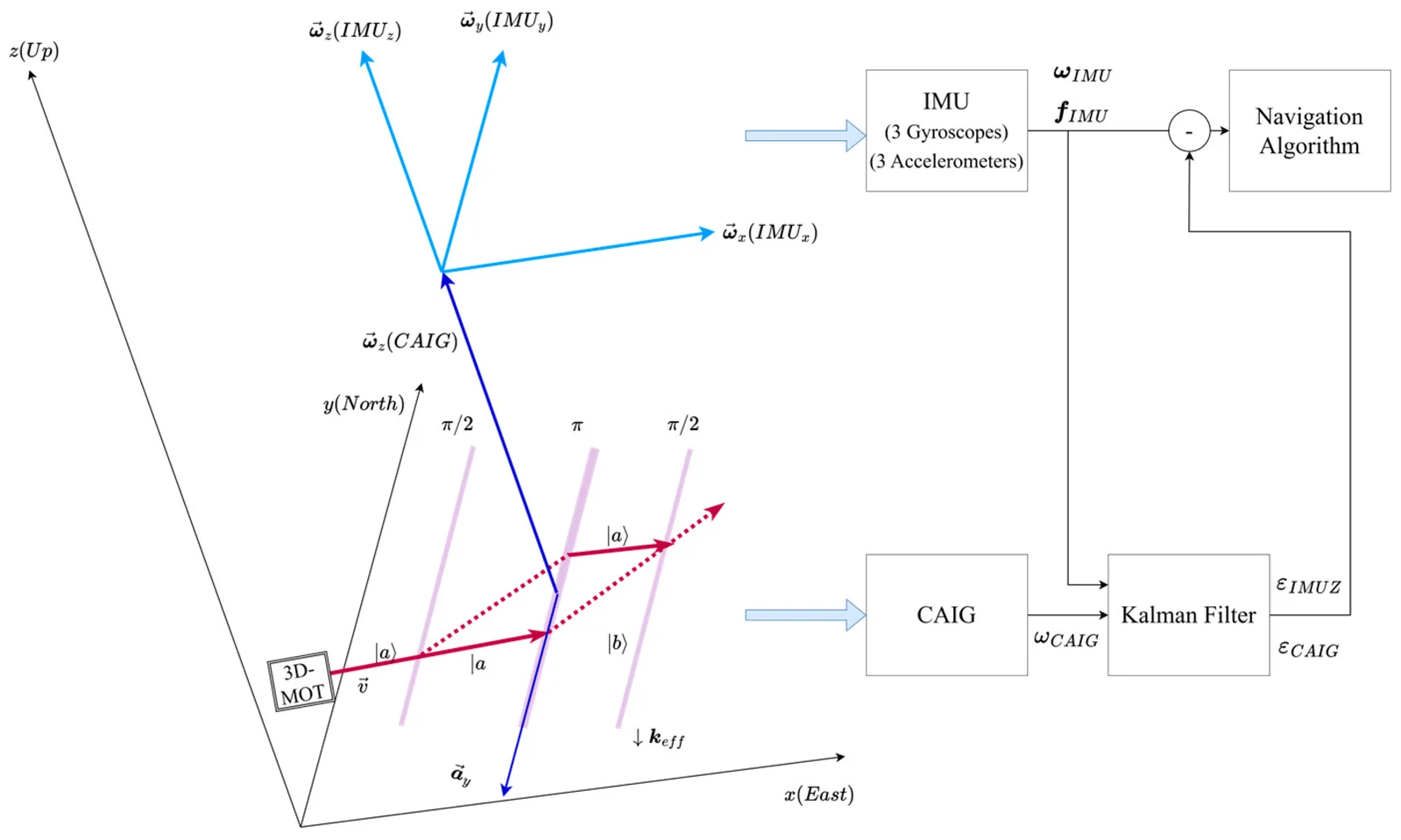
Principle of the CAIG-IMU hybrid inertial navigation system. The left part illustrates the physical sensing principle of CAIG. The conventional IMU and CAIG share the z-axis. The x-axis, y-axis, and z-axis represent the east, north, and up directions, respectively. The right part shows the data-flow architecture of the Kalman filter.

In this paper, a hybrid inertial navigation system is constructed based on a three-pulse opposite projection configuration CAIG and a conventional IMU. Figure 1 illustrates the overall architecture of the CAIG-IMU hybrid inertial navigation system. The system comprises a single-axis CAIG and a three-axis IMU. The IMU consists of three orthogonal gyroscopes and accelerometers, serving as the primary device for inertial navigation updating. The navigation frame is defined as the east–north–up (ENU) coordinate system. The CAIG and IMU share the same sensitive z-axis between the two sensor frames.

The system workflow is also illustrated in Figure 1. In this architecture, the CAIG acts as a high-precision auxiliary sensor to calibrate the z-axis bias of the conventional IMU. The IMU provides high-frequency inertial measurements, including the angular rate and specific force. The dual-bias joint Kalman filter fuses observation data from the IMU and CAIG to estimate the biases of both sensors. The estimated biases are compensated for the raw IMU angular rate data before navigation calculation.

### 2.2. Dual-Bias Estimation Model

It is well known that the accuracy of the INS is limited by the biases of the IMU. For the CAIG-IMU hybrid inertial navigation system described in Section 2.1, the true rotation rate in the z-axis can be formulated as follows:

(1) $$\begin{array}{c} \omega_{trueZ}={\hat{\omega }}_{CAIG}-\varepsilon_{CAIG}={\hat{\omega }}_{IMUZ}-\varepsilon_{IMUZ} \end{array}$$

where ω is the true value of the rotation rate, $\hat{\omega }$ corresponds to measurements of the rotation rate from the conventional IMU and CAIG, and ε is the gyroscope bias. Equation (1) represents the relationships between measured and true rotation rates for the conventional IMU and CAIG, respectively.

On the assumption that the CAIG exhibits negligible bias, the CAIG is treated as the reference sensor, which artificially constrains the CAIG bias to zero, i.e., $\varepsilon_{CAIG}\equiv 0$. Then only the bias errors of the conventional gyroscope are estimated, forming a one-bias estimation model. The state equation only contains the bias state of the conventional gyroscope in z-axis:

(2) $$\begin{array}{c} x(t)=\varepsilon_{IMUZ} \end{array}$$

The observation equation is established based on the angular velocity difference between the two gyroscopes. The observed information is only adopted to correct the errors of the conventional gyroscope, and the corresponding observation model can be expressed as follows:

(3) $$\begin{array}{c} {\hat{\omega }}_{IMUZ}-{\hat{\omega }}_{CAIG}=\varepsilon_{IMUZ} \end{array}$$

Therefore, the observation equation of the Kalman filter for the hybrid navigation system is formulated as:

(4) z(t)=H(t)x(t)+v(t)

where $z(t)={\hat{\omega }}_{IMUZ}-{\hat{\omega }}_{CAIG}$ is the observation vector, $H(t)=I_{1\times 1}$ is the measurement matrix, and v(t)=ε(t) represents the measurement noise vector.

This modeling scheme essentially adopts a single-state and single-observation estimation architecture that heavily relies on the drift-free assumption of the CAIG. However, the CAIG is not an ideal sensor in practical operating scenarios and exhibits weak long-term slow drift. Consequently, the drift-free assumption no longer holds, and the actual observation vector conforms to the following formula:

(5) $$\begin{array}{c} \begin{array}{c} z(t)={\hat{\omega }}_{IMUZ}-{\hat{\omega }}_{CAIG}=\varepsilon_{IMUZ}-\varepsilon_{CAIG}+v(t) \end{array} \end{array}$$

Compared with Equation (3), it can be found that the single-state model artificially ignores the bias of the CAIG and fully attributes the relative bias error between the two gyroscopes to the bias state of the conventional gyroscope. This error coupling effect causes the CAIG drift to be coupled into the conventional IMU bias estimation results, thereby inducing systematic modeling errors [32].

This paper proposes a dual-bias estimation model, which takes the bias of the conventional IMU $\varepsilon_{IMUZ}$ and the bias of the CAIG $\varepsilon_{CAIG}$ as the core states to be estimated:

(6) $$\begin{array}{c} \begin{array}{c} x(t)={\left[\;\begin{array}{cc} \varepsilon_{IMUZ} & \varepsilon_{CAIG} \end{array}\right]}^{T} \end{array} \end{array}$$

The biases of both types of gyroscopes are modeled as continuous random walk processes, and the differential equation of the state is expressed as follows:

(7) $$\begin{array}{c} \dot{x}(t)=\omega (t)={\left[\;\begin{array}{cc} \omega_{\varepsilon_{IMUZ}} & \omega_{\varepsilon_{CAIG}} \end{array}\right]}^{T} \end{array}$$

where ω(t) represents the process noise vector and should satisfy the Gaussian white noise distribution characteristics required for Kalman filtering.

The continuous time state equation is discretized to obtain the state update equation in the discrete time domain:

(8) $$X_{k}=\Phi_{k/k-1}X_{k-1}+W_{k-1}$$

where the state transition matrix is an identity matrix, $\Phi_{k/k-1}=I_{2\times 2}$.

Under the static base condition, the true inertial angular velocity of the carrier is approximately zero. The outputs of the two gyroscopes are dominated by sensor drift and random noise. Based on the dual-gyro observation architecture, a two-dimensional observation equation is constructed to correspond to the measurement outputs of both gyroscopes simultaneously:

(9) $$Z_{k}=HX_{k}+V_{k}$$

where $Z_{k}={\left[\begin{array}{cc} {\hat{\omega }}_{IMUZ} & {\hat{\omega }}_{CAIG} \end{array}\right]}^{T}$ is the observation vector composed of gyroscope observation outputs, $H=\left[\begin{array}{cc} 1 & 0\\ 0 & 1 \end{array}\right]$ is the observation matrix, and $V_{k}$ is the observation noise vector. Traditional filtering treats the observation noise as pure white Gaussian with fixed covariance R. This processing manner is invalid in the presence of non-white noise. Therefore, an adaptive strategy is introduced to realize dynamic correction. Note that the observability analysis of the dual-bias estimation model under static conditions is further discussed in Section 4.4, together with simulation and experimental results.

### 2.3. Adaptive Kalman Filtering Algorithm

The standard discrete Kalman filter (KF) includes prediction and update stages. The formulas for state prediction and covariance prediction are [33]:

(10) $$\begin{array}{c} {\hat{X}}_{k/k-1}=\Phi {\hat{X}}_{k-1/k-1} \end{array}$$

(11) $$P_{k/k-1}=\Phi P_{k-1/k-1}\Phi^{T}+Q$$

The state update and covariance update formulas are given as:

(12) $$\begin{array}{c} K_{k}=P_{k/k-1}H^{T}(HP_{k/k-1}H^{T}+R_{k}{)}^{-1} \end{array}$$

(13) $${\hat{X}}_{k/k}={\hat{X}}_{k/k-1}+K_{k}e_{k}$$

(14) $$P_{k/k}=(I-K_{k}H)P_{k/k-1}$$

The core prerequisite for the validity of the classical Kalman filter is that both the system process noise and measurement noise are zero-mean pure white Gaussian noise without temporal correlation. Noise samples at any time instant are independent of historical data with constant covariance values. Accordingly, the fixed-parameter Kalman filter adopts a constant measurement noise covariance to characterize measurement confidence. However, extensive studies on inertial sensor noise modeling have validated that the random drifts of fiber-optic gyroscopes and cold atom interferometric gyroscopes do not follow the statistical properties of independent and identically distributed white noise [29,34].

To address this problem, a residual autocorrelation-based adaptive Kalman filtering algorithm is proposed, which can dynamically estimate the measurement noise covariance matrix for online Kalman filtering. By correcting the measurement noise covariance in real time and dynamically optimizing measurement confidence, the algorithm further improves the stability and accuracy of state estimation on the basis of the dual-bias estimation model. This achieves dual improvements brought by model structural optimization and algorithm adaptation.

To accurately quantify noise intensity, this paper constructs a residual autocorrelation identification model based on the sliding window time series statistical method, so as to capture the time varying and temporally correlated characteristics of noise in real time. The Kalman filter innovation residual sequence is defined as the difference between measurements and predicted observations:

(15) $$\begin{array}{c} e_{k}=Z_{k}-H{\hat{X}}_{k/k-1} \end{array}$$

A sliding window with fixed length is adopted to extract segments of the innovation residual sequence, where the window length is L. The residual samples within the window are normalized, and the first-lag autocorrelation coefficient is calculated to quantify the temporal-correlation strength of colored noise:

(16) $$\rho_{k}=\frac{Cov(e_{k-L+1}:e_{k-1},e_{k-L+2}:e_{k})}{\sqrt{Var(e_{k-L+1}:e_{k-1})}\sqrt{Var(e_{k-L+2}:e_{k})}}$$

where $\rho_{k}\in$[−1,1] denotes the real-time residual autocorrelation coefficient for quantifying the temporal-correlation strength of noise, Cov(⋅) represents the covariance of residual samples inside the window; and Var(⋅) is the variance of the residual sequence. When $\left|\rho_{k}\right|$ approaches 1, the residual exhibits a stronger temporal tailing effect, the temporal correlation of noise becomes more prominent, and the model mismatch of conventional fixed-parameter filtering turns more severe. When $\left|\rho_{k}\right|$ approaches 0, the residuals approximately satisfy the uncorrelated property of pure-white noise, indicating good matching performance of the filtering model.

The time-varying measurement-noise covariance updating model driven by the autocorrelation coefficient is expressed as:

(17) $$\begin{array}{c} R_{k}=R_{0}\cdot (1+\lambda \cdot |\rho_{k}|) \end{array}$$

where $R_{0}$ is the initially calibrated measurement noise covariance; λ denotes the adaptive adjustment coefficient, which regulates the sensitivity of parameter updating to avoid excessive or invalid parameter iteration; $\rho_{k}$ is the real-time residual autocorrelation coefficient, serving as the sole criterion for judging time-varying noise characteristics. Under steady noise conditions with negligible residual autocorrelation, $|\rho_{k}|\approx 0$ and $R_{k}\approx R_{0}$, the algorithm retains the basic measurement constraints to guarantee state estimation accuracy. When colored noise dominates and residual temporal correlation increases, $\left|\rho_{k}\right|$ rises accordingly, leading to an adaptive increase to suppress filter drift induced by colored noise. Meanwhile, upper and lower threshold limits of $R_{k}$ are predefined to constrain the updating range of measurement noise covariance. This setting prevents abnormal filter gain or convergence failure caused by excessive parameter updating, and ensures the stability and reliability of long-term iterative filtering from the perspective of parameter constraint.

## 3. Simulation Analysis

The hybrid navigation system is theoretically validated via numerical simulations in this section, with experimental verification provided in Section 4. Simulation experiments are conducted to verify the model and algorithm, covering noise characteristics analysis, model investigation, algorithm effectiveness evaluation and integrated navigation error calculation.

**Figure 2:**
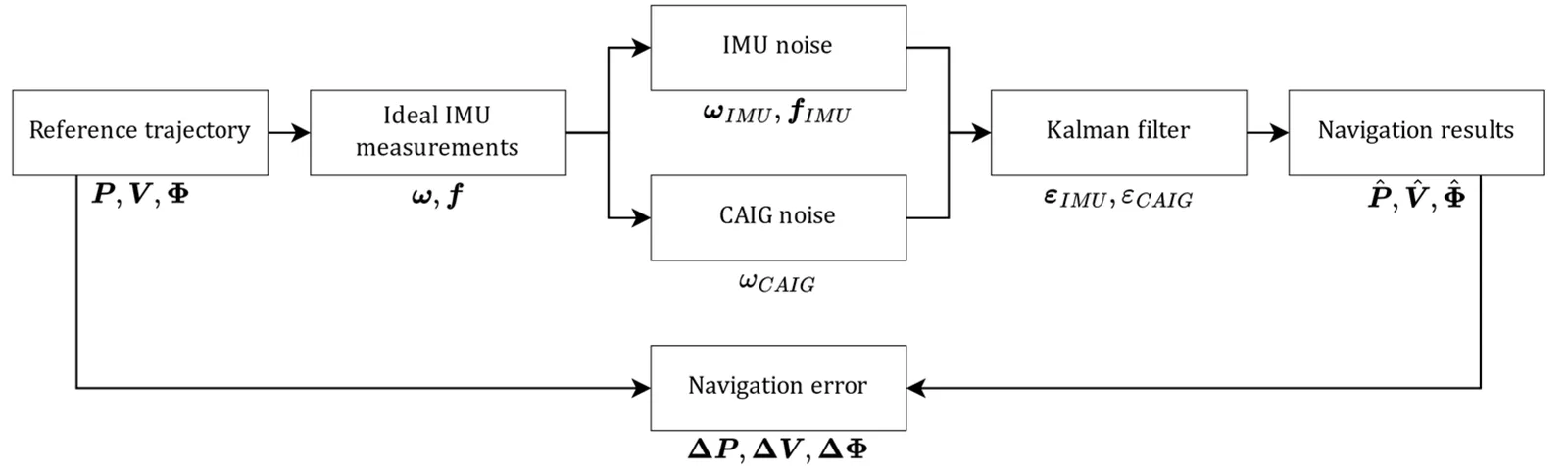
Block diagram of the numerical simulation. The reference trajectory provides true navigation quantities including position, velocity and attitude to generate ideal IMU measurements. Sensor noise is added to obtain the noisy measurements of conventional IMU and CAIG. The dual-bias Kalman filter takes these sensor outputs as inputs and yields the integrated navigation error.

The simulation workflow is shown in Figure 2. The true position, velocity and attitude provided by the reference trajectory are used to generate ideal inertial measurements. In the simulations, a static reference trajectory is adopted, i.e., the position, velocity, and attitude of the platform remain unchanged, with the reference coordinates set to the local geodetic coordinates. Sensor noise is added to produce noisy outputs for the conventional IMU and the single-axis CAIG. The sensor noise considered in the simulations consists of three components [34]: (i) the constant bias, (ii) the angle random walk (ARW) white noise, and (iii) a non-stationary time-varying noise generated by a first-order autoregressive AR(1) model with coefficient 0.97. The ARW intensities of the conventional IMU and the CAIG are determined according to the typical noise performance of the corresponding sensors. The bias and the ARW are listed in Table 1. The noisy sensor measurements are fed into the dual-bias Kalman filter to jointly estimate both IMU and CAIG gyro biases. Finally, the navigation error ΔP is obtained to evaluate the filtering performance.

In the simulation, the error-free rotation rate and acceleration measurements are generated using the reference motion trajectory P,V,Φ of the carrier based on the inverse mechanization of the navigation algorithm [35]. Based on the performance of inertial sensors, the noisy measurements of the rotation rate and acceleration can be obtained by introducing biases and noises of the conventional IMU and CAIG. As described in Section 2, the corrected rotation rate can be obtained using the Kalman filter. Subsequently, the raw and corrected rotation rate and acceleration are fed into the navigation mechanization to calculate navigation solutions. Thus, navigation errors can be acquired by comparing the solved results with the predefined reference trajectory. The simulation initial geodetic coordinates are set to a latitude of 30.395° N and a longitude of 114.348° E. The inertial sensor parameters including bias, angle random walk (ARW), and the data output rate are listed in Table 1.

### 3.1. Gyroscope Noise Simulation

**Figure 3:**
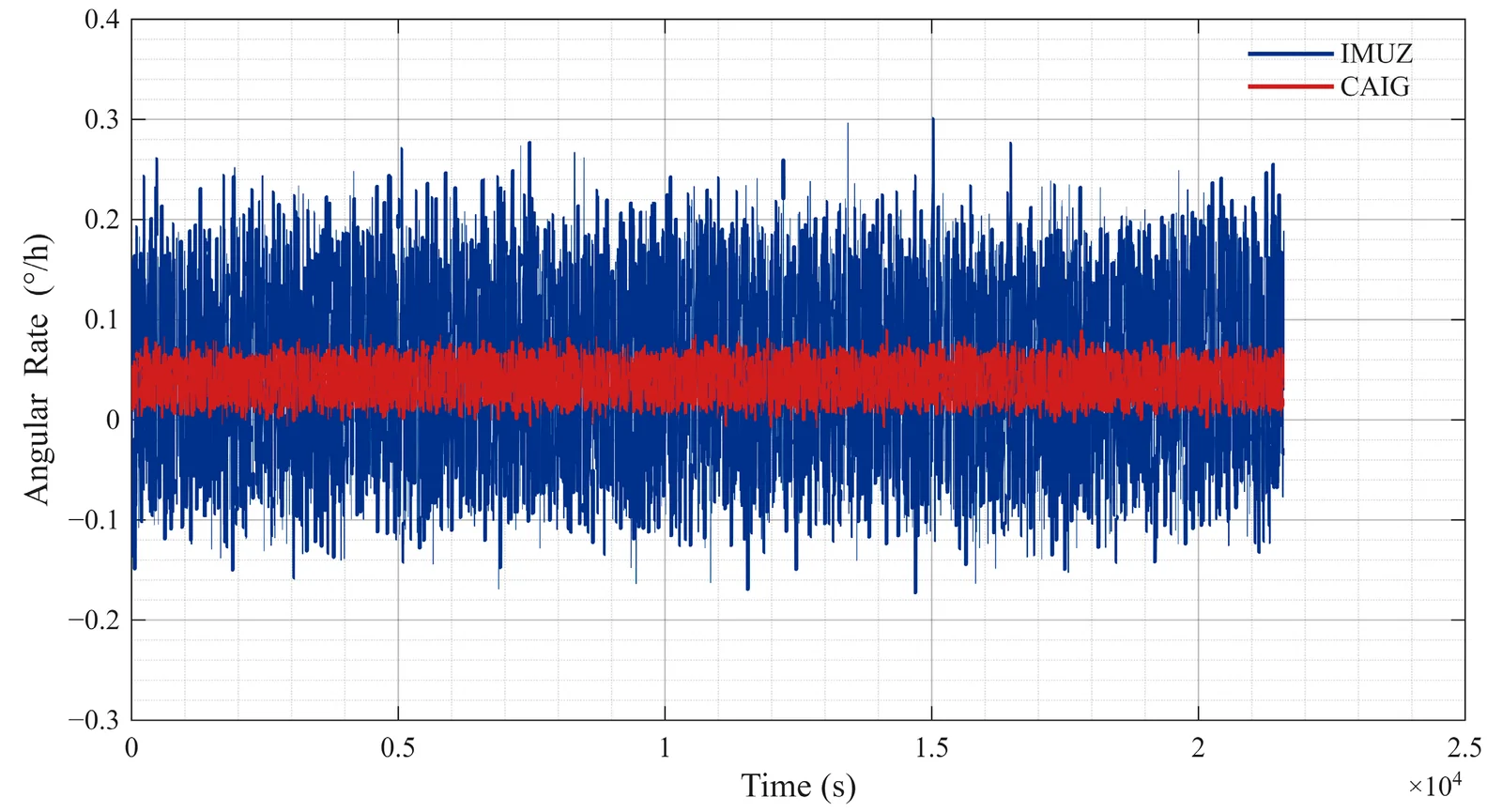
The simulated measurements of the conventional IMU in z-axis (blue line) and the CAIG (red line).

We first perform simulation experiments to analyze gyroscope noise characteristics. The true bias parameters of gyroscopes are preset, where the true bias of the conventional gyroscope is $0.05^{\circ }/h$, and the true bias of the CAIG is $0.04^{\circ }/h$. Under these conditions, time-varying noise is added to emulate the raw sensor output characteristics. As shown in Figure 3, the angular-rate measurements about the z-axis are composed of the fixed bias values, the ARW noise and the time-varying noise. It can be observed that the IMU exhibits larger noise amplitude compared with the CAIG.

**Figure 4:**
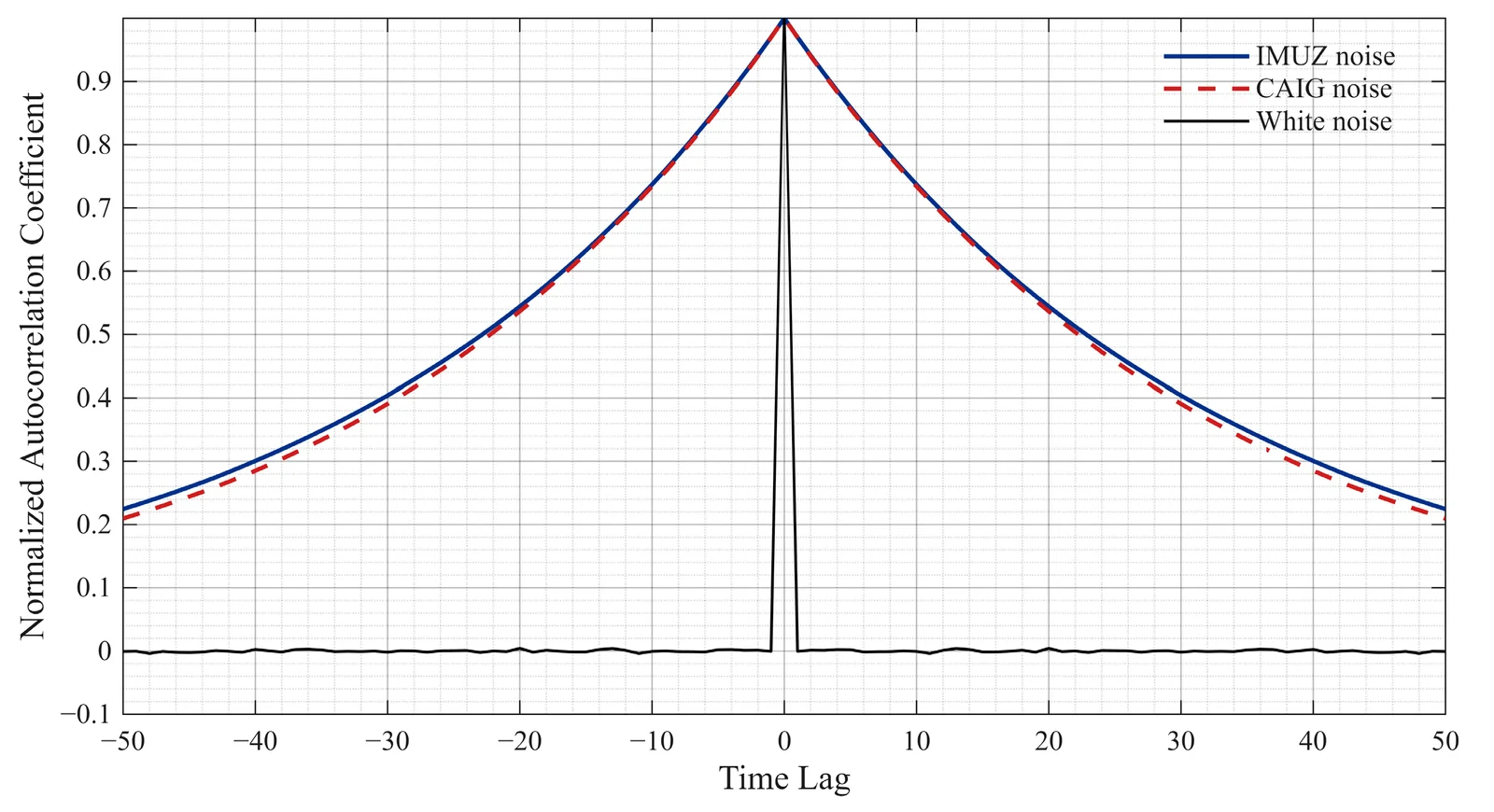
The normalized autocorrelation coefficients of the conventional IMU (blue line), the CAIG (red line) and the white noise (black line).

Normalized autocorrelation analysis is performed for the two sets of measurements, as illustrated in Figure 4. Before the autocorrelation analysis, the constant bias terms are removed from the measurement sequences, so that the autocorrelation statistics reflect the pure noise components rather than the constant offsets. The autocorrelation curves demonstrate that gyroscope noise shows a tailing effect and temporal correlation, which deviates from ideal pure white Gaussian noise. The normalized autocorrelation coefficients of gyroscope noise remain at approximately 0.22 at a lag of 50, which agrees well with the theoretical value of the AR(1) model (0.97^50^ ≈ 0.218), revealing prominent temporal-memory effects. By contrast, for Gaussian white noise, the normalized autocorrelation coefficient decays below 0.01 within two lags with no visible tailing effect, satisfying the independent and identically distributed property. Theoretically, the autocorrelation coefficient of white noise should be significant only at zero lag and decay rapidly toward zero for all other lag orders. However, the autocorrelation curves of gyroscope noise decay slowly, and correlation persists over multiple lags. This behavior conforms to the temporal evolution characteristic of first-order autoregressive noise.

### 3.2. Gyroscope Bias Estimation Simulation

**Figure 5:**
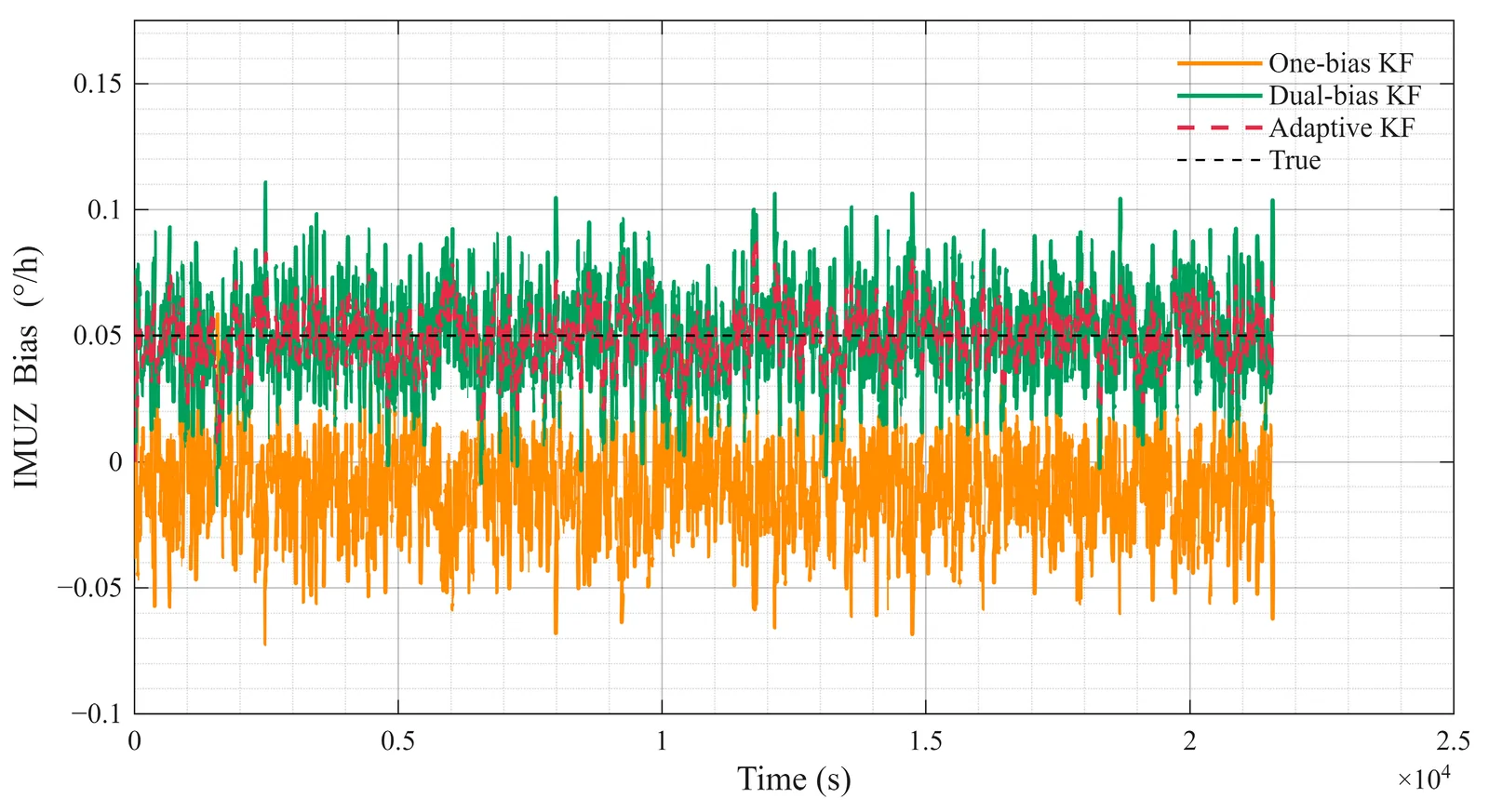
Estimated gyroscope bias of the conventional IMU Z-axis. The orange line denotes the result of the one-bias KF, the green line represents the result of dual-bias KF with fixed measurement covariance, and the red dashed line corresponds to the adaptive KF. The black dashed line represents the true value.

Based on the simulated measurements of the conventional IMU and the CAIG, the Kalman filter is adopted to estimate the biases of the conventional IMU according to the estimation model depicted in Section 2. The filter initial parameters are set with the initial state vector as zero, the initial error covariance as 0.003 $({}^{\circ}/h{)}^{2}$, the process noise covariance as 1 × 10^−9^ $({}^{\circ}/h{)}^{2}$, and the initial measurement noise covariance as 5 × 10^−4^ $({}^{\circ}/h{)}^{2}$. For the adaptive algorithm, the sliding-window length is 100 and the adjustment coefficient is 7.2. The bias estimation results of the conventional IMU in the z-axis are presented in Figure 5. Three comparative filtering schemes are conducted in the simulation tests.

The first one is the single bias estimation model with fixed measurement covariance, denoted as one-bias KF. The second is the dual-bias estimation model with fixed measurement covariance, denoted as dual-bias KF. The third combines the dual-bias estimation model with the adaptive measurement covariance, denoted as adaptive KF. For the one-bias KF, the observation vector is constructed from the measurement difference between the conventional IMU and the CAIG; consequently, the filter outputs exhibit obvious estimation deviation. For both dual-bias KF and adaptive KF, the observation vectors are formed from the raw measurements of the conventional IMU and the CAIG, and their estimated gyroscope biases approach the true value. Owing to the adaptive adjustment of measurement covariance, the adaptive KF achieves the best tracking accuracy and effectively suppresses estimation fluctuation.

It can be observed from the simulation results that the one-bias KF fails to converge to the preset true value of 0.05°/h. The main reason is that, when the differential observation ${\hat{\omega }}_{IMUZ}-{\hat{\omega }}_{CAIG}$ is used to solve for the bias of the conventional IMU, the bias of the CAIG is coupled into the conventional IMU bias estimation results. In contrast, the two types of dual-bias KF achieve improved estimation accuracy compared with the one-bias KF.

To quantitatively compare the bias estimation accuracy of different methods, mean error, relative error and standard deviation are computed under the same simulation framework with identical noise conditions. The performance gains brought by model-structure optimization and adaptive filter optimization are quantified respectively. The quantitative comparison results are summarized in Table 2. The calculation formula for relative error is given as:

(18) $$\begin{array}{c} Relative\;error=\frac{\left|Estimation-True\right|}{True}\times 100\% \end{array}$$

As indicated by the quantitative results in Table 2, affected by error coupling and model mismatch, the bias estimation results from the one-bias KF have a relative error as high as 80.4%. The mean of bias estimation 0.0098°/h is highly consistent with the difference between the true values of the two gyroscopes. The dual-bias joint model structurally resolves the error coupling problem and eliminates systematic deviations, reducing the relative error to 2.8%. On the basis of dual-bias KF, the adaptive KF further reduces the standard deviation of bias estimation to 0.0108°/h. These results indicate that the model addresses the error-coupling issue, while adaptive KF further suppresses error fluctuations. The two progressive improvements jointly enhance the accuracy and stability of bias identification.

**Figure 6:**
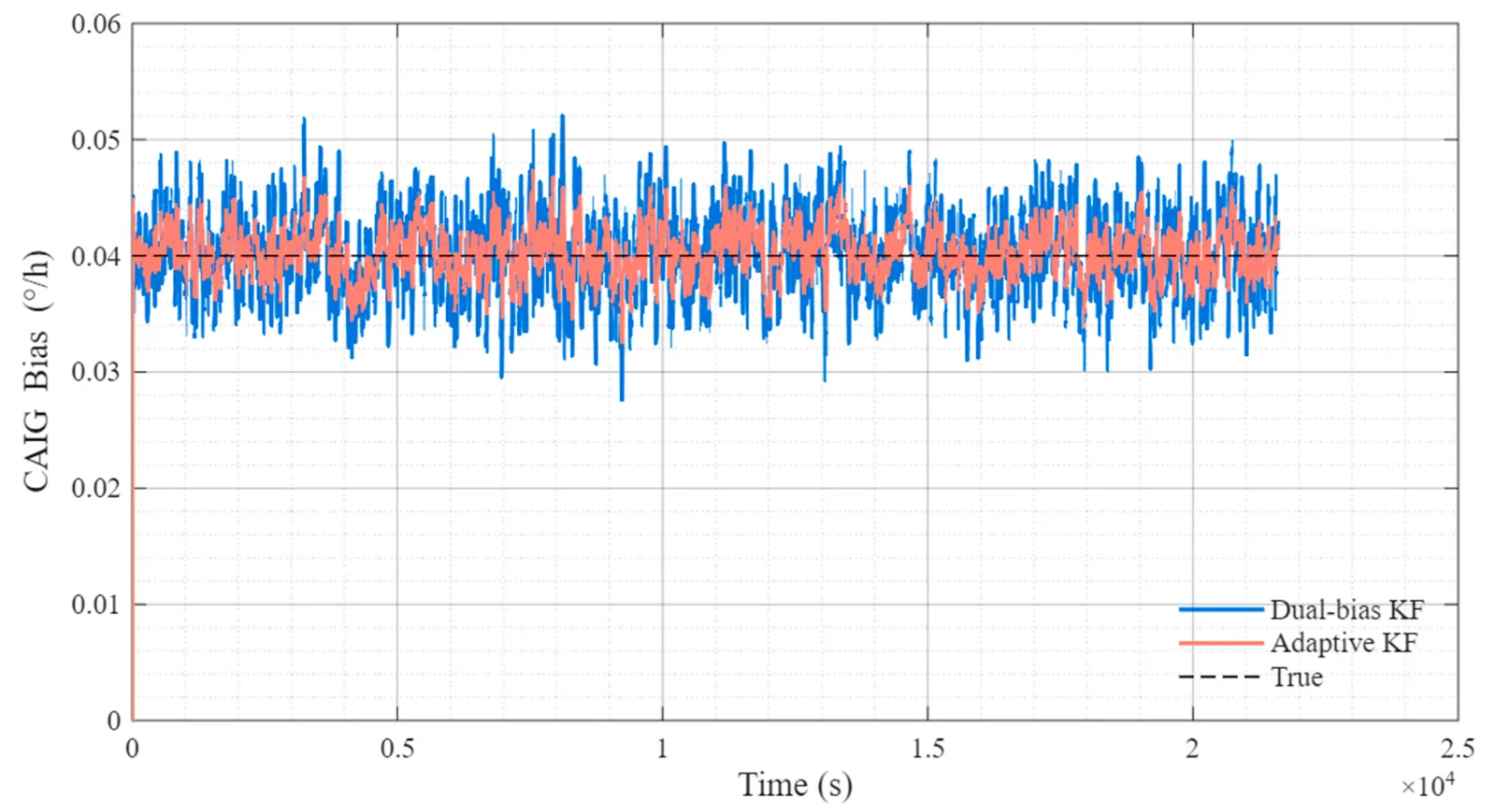
Estimated bias of the CAIG by dual-bias KF (blue line) and adaptive KF (red line).

The dual-bias model enables simultaneous bias estimation for the CAIG, as shown in Figure 6. The black dashed line denotes the true bias of 0.04°/h, the blue curve represents the estimation result of the dual-bias KF, and the red curve represents the estimation result of the adaptive KF. The simulated results reveal that the dual-bias estimation model can effectively decouple the biases of the conventional IMU and the CAIG. The bias estimation curve of the dual-bias KF exhibits large fluctuations with a standard deviation of 0.0033°/h. In comparison, the adaptive KF reduces the standard deviation of the CAIG bias estimation to 0.0020°/h and achieves better agreement with the true value. It should be noted that sufficient prior conditions are available in this simulation, so the CAIG bias can converge toward its true value. In practical static tests, only an approximate estimate of the CAIG bias can be derived from the Earth-rotation component, subject to alignment errors.

### 3.3. Navigation Error Simulation

**Figure 7:**
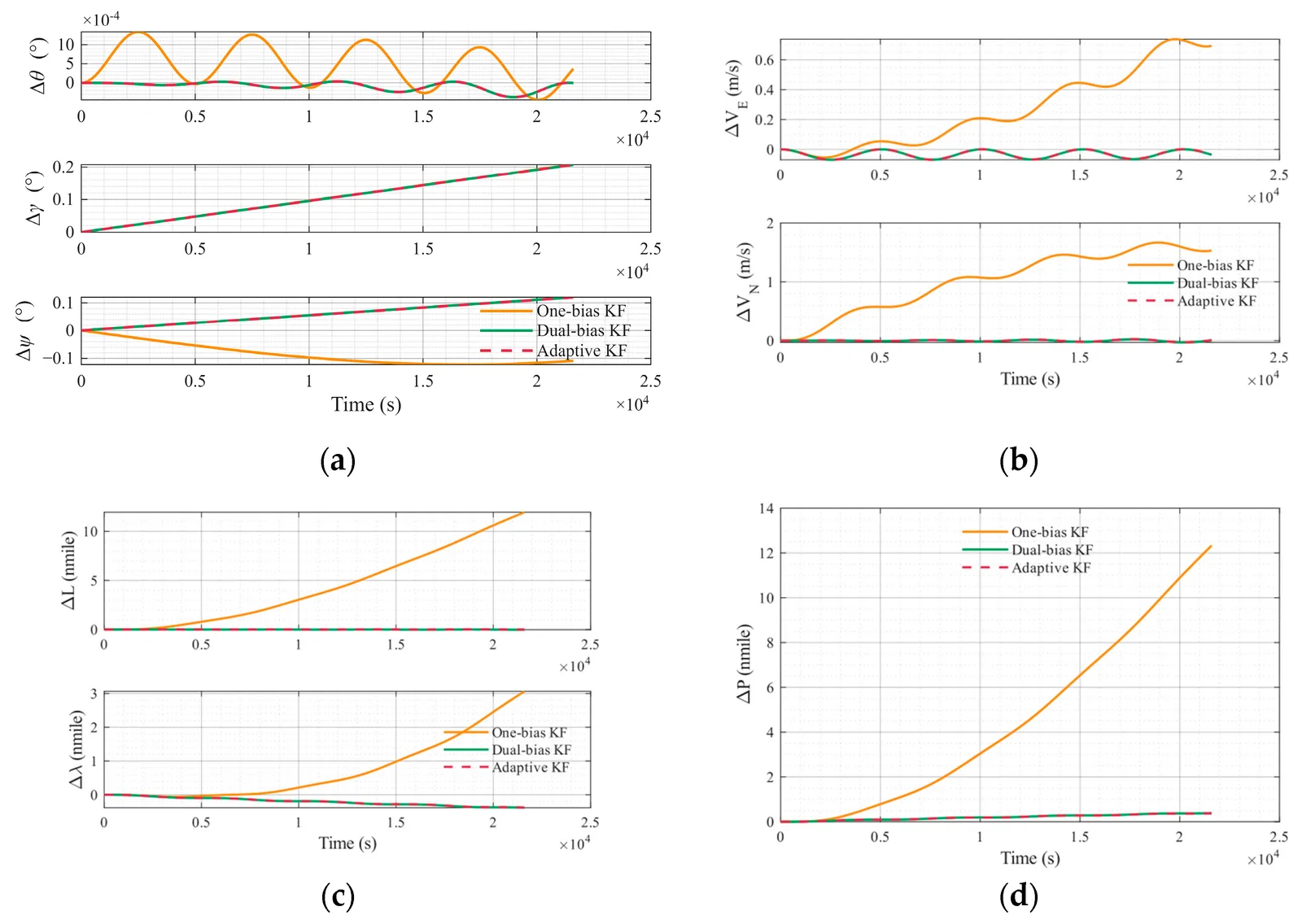
Navigation errors of the single-axis hybrid navigation system. (**a**) The attitude error of pitch, roll, and yaw from top to bottom. (**b**) The velocity error. The first image illustrates east velocity, and the other illustrates north velocity. (**c**) The latitude error is shown in the first image, and the longitude error is shown in the second image. (**d**) The positioning error.

After the Kalman filter has converged, the estimated bias is subtracted from the measurements of the conventional IMU. The corrected measurements are subsequently processed by the navigation algorithm to compute attitude, velocity, and position solutions. The corresponding navigation errors are presented in Figure 7 and Table 3, Table 4 and Table 5. In Figure 7, the results obtained from the one-bias KF, dual-bias KF and adaptive KF are depicted by the yellow, green, and red curves, respectively.

As shown in Figure 7 and Table 3, Table 4 and Table 5, the improvement in the z-axis gyro bias accuracy leads to enhanced precision of the hybrid navigation system. Compared with the one-bias KF, the dual-bias KF and adaptive KF effectively reduce both the velocity error and the position error, with the position error decreased by 96.9% and 97.0%, respectively. However, the attitude accuracy shows only insignificant improvement, especially in the roll and yaw channels. Based on the established simulation framework, the results demonstrate that the dual-bias joint model effectively resolves the static error-coupling issue, yielding remarkable improvements in both bias-identification accuracy and navigation output performance.

To further verify the proposed method under sampling conditions consistent with the actual experimental platform in Section 4, additional multi-rate simulations are conducted with the IMU and CAIG output rates set to 1000 Hz and 0.4 Hz, respectively. The standard deviations of the bias estimation for the one-bias KF, the dual-bias KF, and the adaptive KF are 0.0305°/h, 0.0180°/h, and 0.0129°/h, respectively, with relative errors of 74.2%, 3.5%, and 3.3%. The positioning error is reduced by 93.4% and 93.6% for the dual-bias KF and the adaptive KF compared with the one-bias KF. Compared with the equal-rate simulation, the lower measurement update rate of 2.5 s leads to larger estimation fluctuation and slightly degraded accuracy. Nevertheless, the dual-bias joint modeling still eliminates the systematic bias error, and the adaptive KF further suppresses the estimation fluctuation, confirming the effectiveness of the proposed method under the multi-rate sampling characteristics.

## 4. Experimental Results and Discussion

### 4.1. Static Experimental Platform

**Figure 8:**
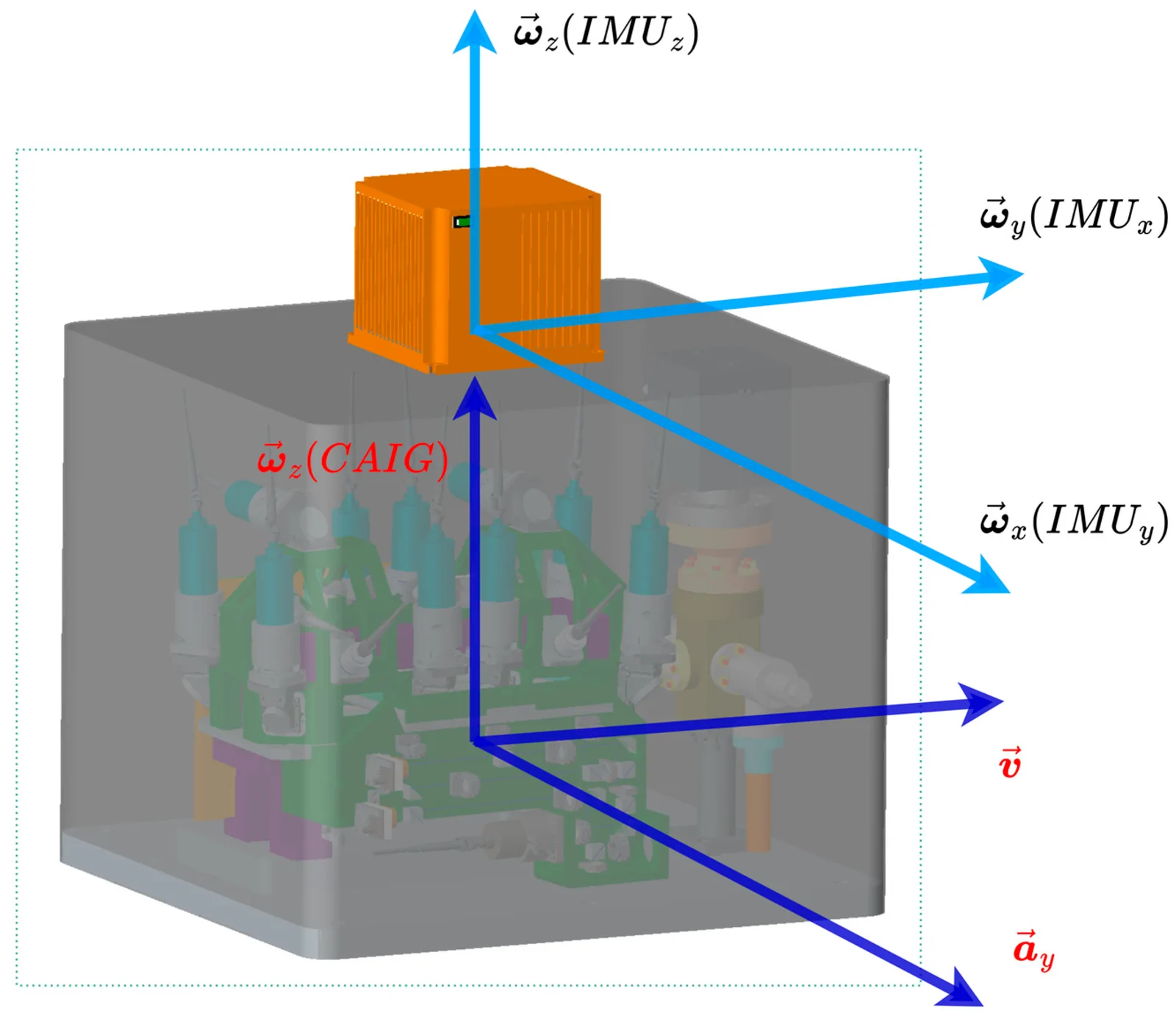
Three-dimensional structural model of the CAIG-IMU hybrid navigation system, consisting of a single-axis CAIG and triaxial fiber-optic IMU.

To further validate the navigation performance, a CAIG-aided fiber-optic IMU experimental platform is constructed for static inertial navigation tests, as shown in Figure 8. The platform primarily comprises a high-precision CAIG, a fiber-optic IMU, an experimental bench, and a data-acquisition device. The self-developed CAIG based on the Sagnac effect adopts a three-pulse counter-propagating configuration as in our previous work [36]. The glass vacuum chamber in the sensor head is mainly separated into three parts: two three-dimensional magneto-optical traps with a recapturing technique and one common interference region. The interference region is illuminated by three parallel, spaced Raman beams, which are created by a set of polarized beam splitters and corresponding wave plates. After the atomic loading procedure, two atom clouds are launched towards each other at velocities of 5 m/s. The atoms are further cooled down using moving molasses and then prepared in the |F = 2, mF = 0⟩ state using optical pumping. An atomic interference sequence consisting of three Raman pulses with a pulse interval of T = 13.8 ms is applied immediately afterward. Finally, a normalized detection technique is used to obtain the rotation phase. The CAIG is capable of measuring the angular rate with a bias stability of 0.002°/h at an integration time of 1000 s. The fiber-optic IMU (Xingguan Technology F71A, Wuhan, China) is equipped with three-axis fiber-optic gyroscopes and quartz accelerometers, with physical dimensions of 130 × 130 × 110 mm^3^. It outputs high-rate inertial measurements at 1000 Hz, while the CAIG provides low-rate, angular-rate measurements at 0.4 Hz. A time-synchronization module is used to achieve timestamp alignment between the two sensors. During the static tests, the entire sensor suite, i.e., the CAIG–IMU hybrid navigation system, is rigidly mounted on the surface of the experimental bench. The experimental data processing was implemented in MATLAB 2025b.

### 4.2. Noise and Bias Estimation Tests

**Figure 9:**
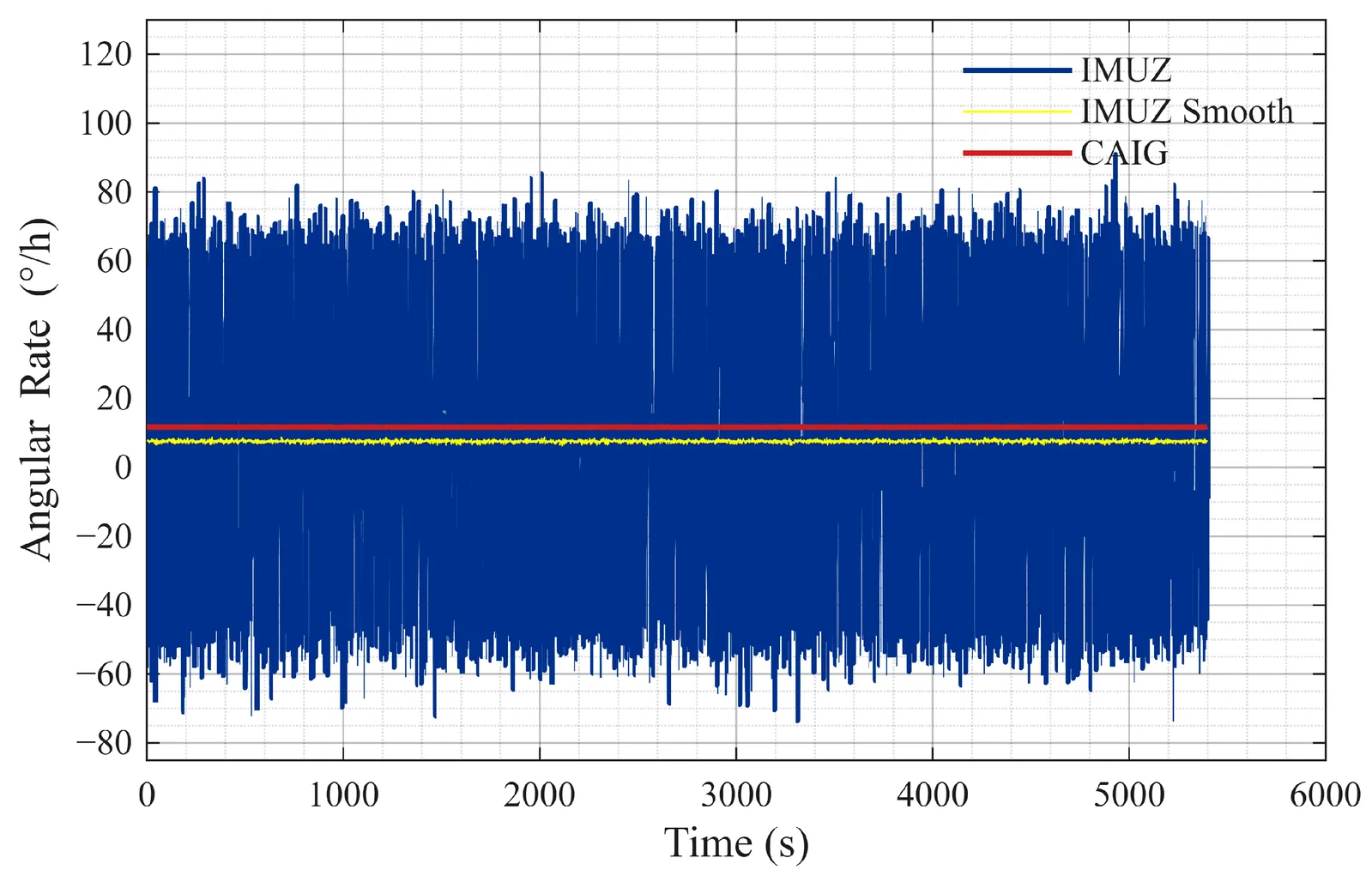
Raw angular-rate observations from IMU Z-axis and CAIG.

The raw angular-rate measurements of the fiber-optic gyroscope and CAIG under static conditions are depicted in Figure 9. The raw output of the fiber-optic gyroscope (blue curve) exhibits large-amplitude high-frequency random fluctuations. After smoothing, the fiber-optic gyroscope output (yellow curve) effectively suppresses the high-frequency noise components, revealing the underlying bias. The smoothing is performed using a moving average filter with a window length of 2000 samples. Note that the smoothing is applied only for visualization purposes, and the subsequent bias estimation and noise analyses are based on the original unsmoothed measurements. In comparison, the CAIG output (red curve) is much smoother, with lower noise amplitude. A constant offset exists between the static outputs of the two sensors. This offset originates jointly from the biases of the fiber-optic gyroscope and the CAIG, and it cannot be separated from the observation of a single sensor. By applying the dual-bias estimation model to the measured dataset, the navigation performance under real-world noise conditions can be evaluated.

**Figure 10:**
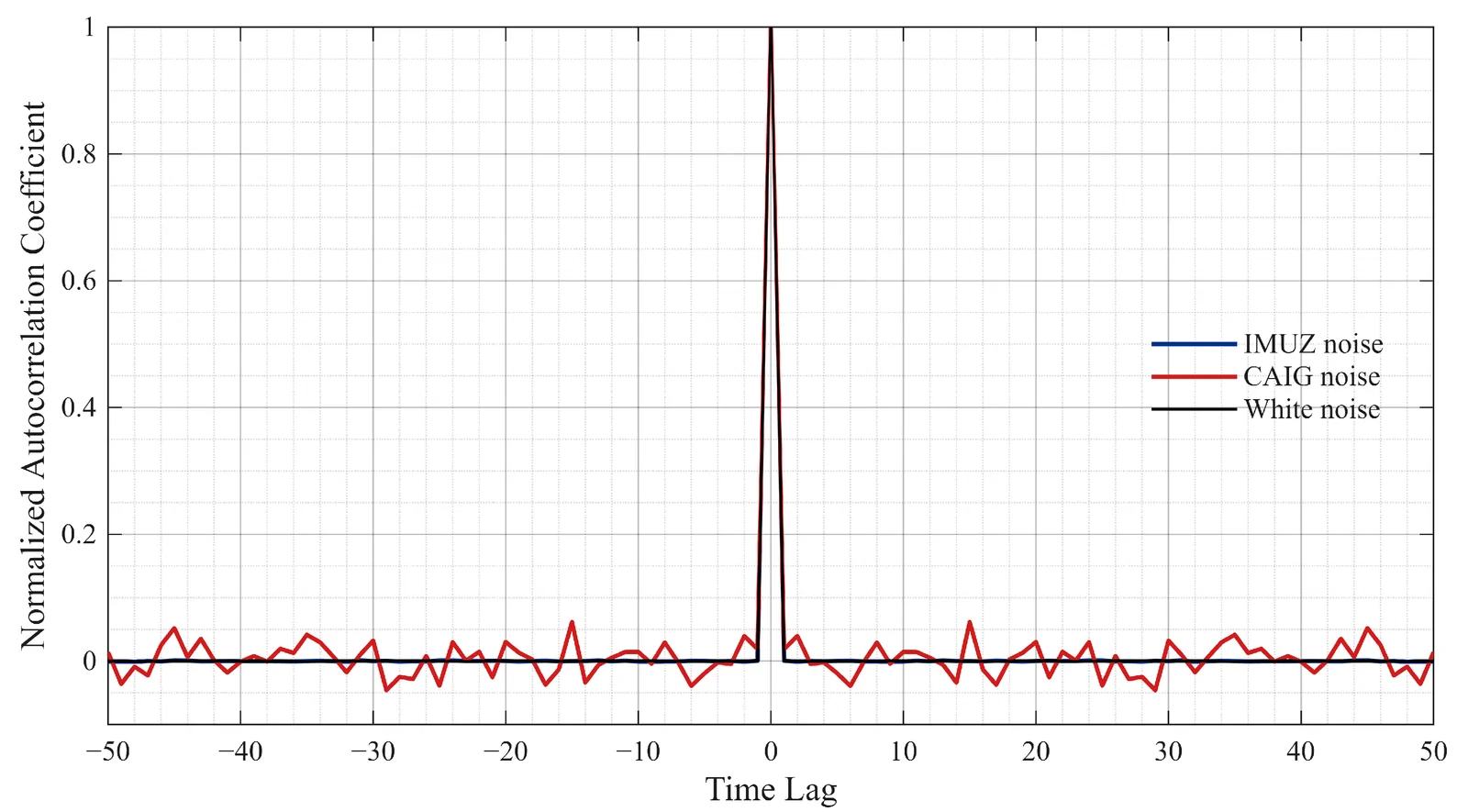
Normalized autocorrelation coefficient of gyroscope measurements and white noise versus time lag.

**Figure 11:**
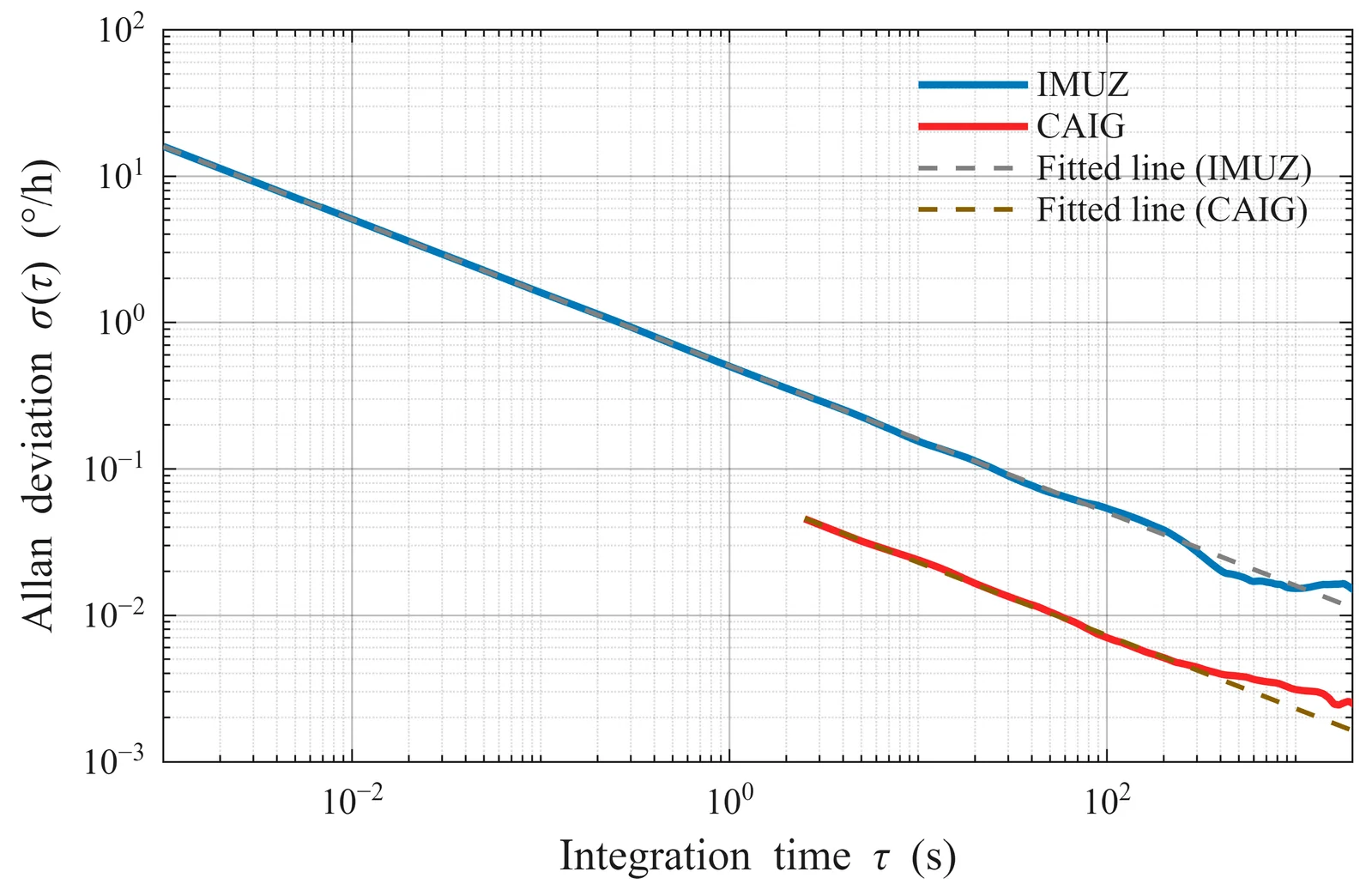
Allan deviation analysis for measured data of fiber-optic gyroscope and cold-atom interferometer gyroscope.

Figure 10 presents the normalized autocorrelation results of the measured noise sequences from the fiber-optic gyroscope and the CAIG, with white noise provided as a reference. After mean removal, the autocorrelation coefficients of both gyroscope noises decay rapidly to near zero within 50 lags, exhibiting a sharp peak only at zero lag and dominated by the angle random walk component (ARW). It should be noted that the CAIG outputs at a low rate of 0.4 Hz, and the limited number of available samples results in statistical fluctuations in the estimated autocorrelation coefficients. The low-frequency time-correlated components, which are not resolved within such short lags, are revealed by the Allan deviation analysis in Figure 11, where both curves gradually deviate from the ARW-fitting lines at long integration times.

The log–log Allan deviation curves [37] of measured outputs from the two gyroscopes are illustrated in Figure 11. The red curve denotes the fiber-optic gyroscope, and the blue curve corresponds to the CAIG, while the gray lines represent theoretical fitting references. The bias stability of the fiber-optic gyroscope is 0.05°/h at an integration time of 100 s, whereas the CAIG achieves a bias stability of 0.002°/h at 1000 s. At short integration times, both curves exhibit the slope characteristic typical of angle random walk (ARW). As the integration time increases, the Allan deviation curves gradually deviate from the ARW-fitted lines and exhibit trends associated with slow-varying drift, demonstrating the existence of slowly varying noise components in gyroscope outputs. Comparison shows that the Allan deviation magnitude of the CAIG is lower than that of the fiber-optic gyroscope at identical integration times, indicating superior noise performance of the CAIG and its potential benefit to the hybrid navigation system.

**Figure 12:**
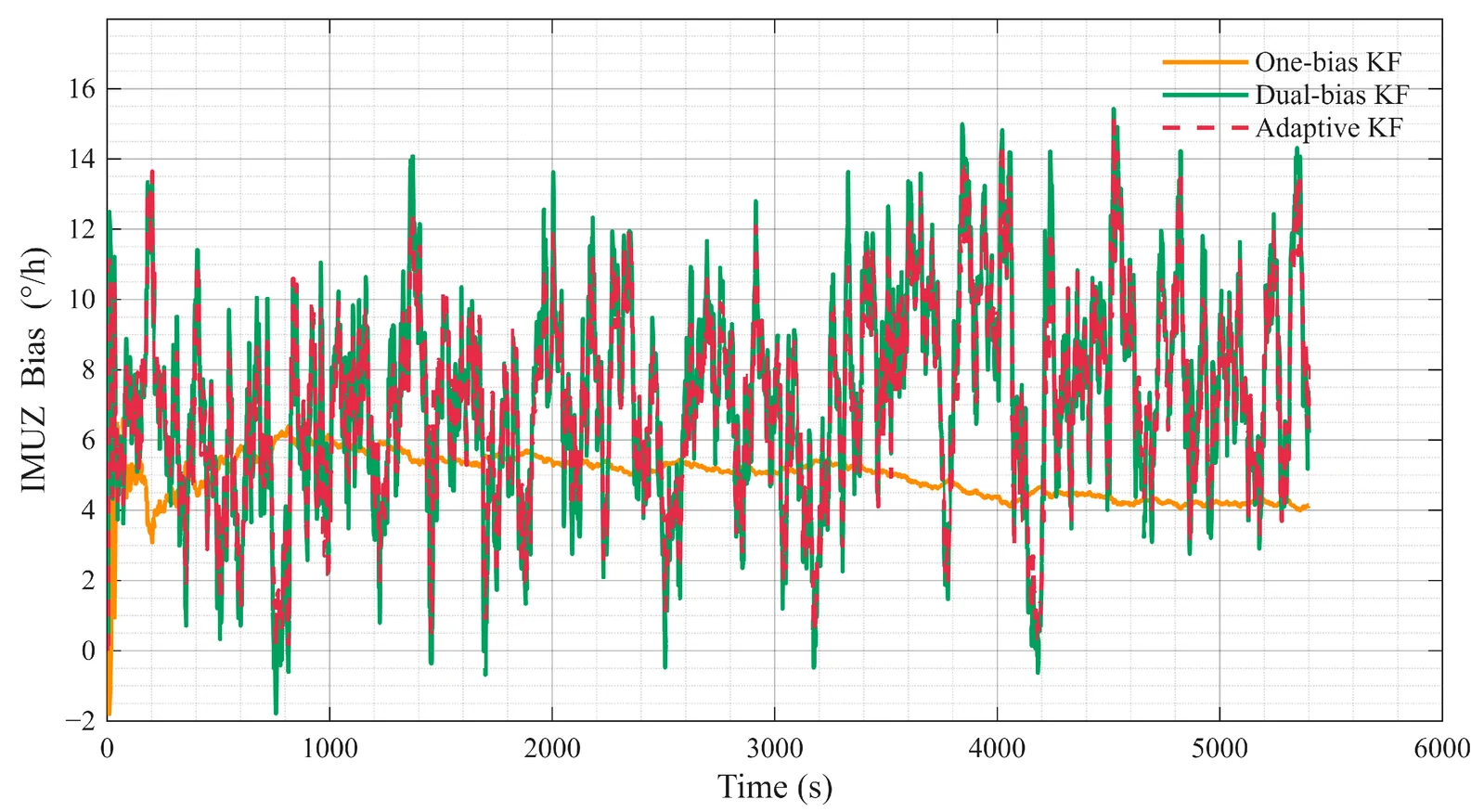
Estimated gyroscope bias of the conventional IMU Z-axis. The orange line denotes the result of the one-bias KF, the green line is the result of the dual-bias KF with fixed measurement covariance, and the red dashed line corresponds to the adaptive dual-bias KF.

Figure 12 shows the bias estimation results of the conventional IMU Z-axis obtained by the three filtering schemes using 5400 s of static measurement data. The orange curve corresponds to the one-bias KF, the green curve represents the dual-bias KF, and the red curve is obtained by the adaptive KF. The dual-bias KF estimation sequence exhibits large fluctuations due to the large-amplitude noise of the fiber-optic gyroscope. The adaptive KF partially suppresses the noise-induced fluctuations compared with the dual-bias KF.

**Figure 13:**
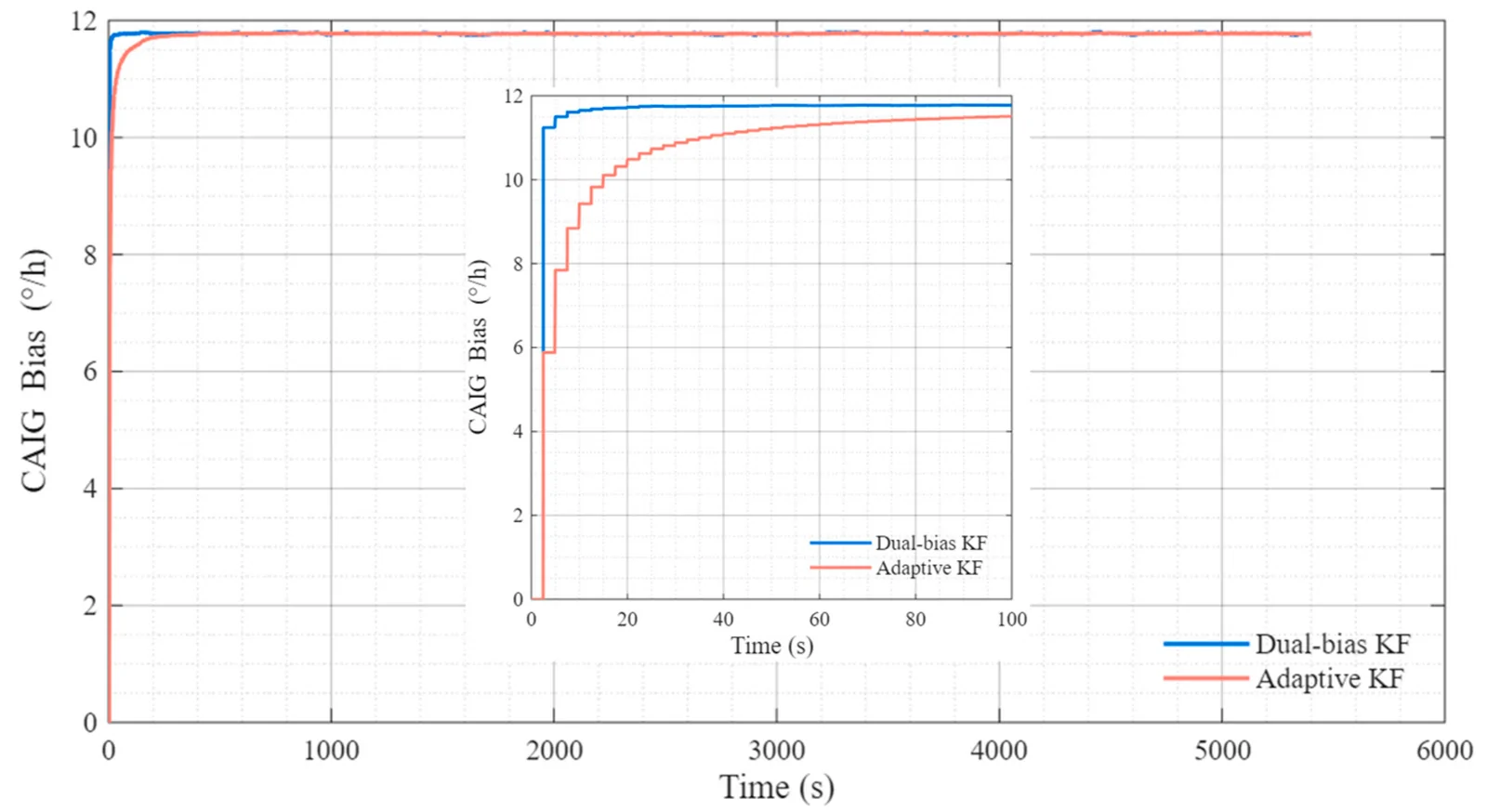
CAIG bias estimation results of the dual-bias KF and the adaptive KF based on static measured data. The inset shows the enlarged convergence stage within the first 100 s.

The CAIG bias estimation results obtained by dual-bias KF and adaptive KF are presented in Figure 13. Both algorithms converge to the same steady-state level within 100 s. The inset plot provides a magnified view of the initial filtering stage, where we can see the update time is 2.5 s. The dual-bias KF reaches its steady state faster, whereas the adaptive KF exhibits a slightly slower but smoother convergence, owing to the real-time adjustment of the measurement covariance. The time difference to reach the steady-state is negligible for long-duration inertial navigation systems. After convergence, the adaptive KF shows smaller fluctuations than the dual-bias KF. After reaching a steady state, the two estimation curves are close to each other, which demonstrates that the adaptive scheme does not degrade the steady-state estimation capability of the dual-bias joint model. The steady-state CAIG bias estimated by the filter (11.8°/h) is consistent with the mean of the raw static measurements. Subtracting the vertical component of the Earth’s rotation (approximately 7.6°/h) from this value yields an approximate estimate of the absolute CAIG bias, subject to alignment errors. A more accurate verification of the absolute bias can be achieved by turntable calibration.

### 4.3. Navigation Results

**Figure 14:**
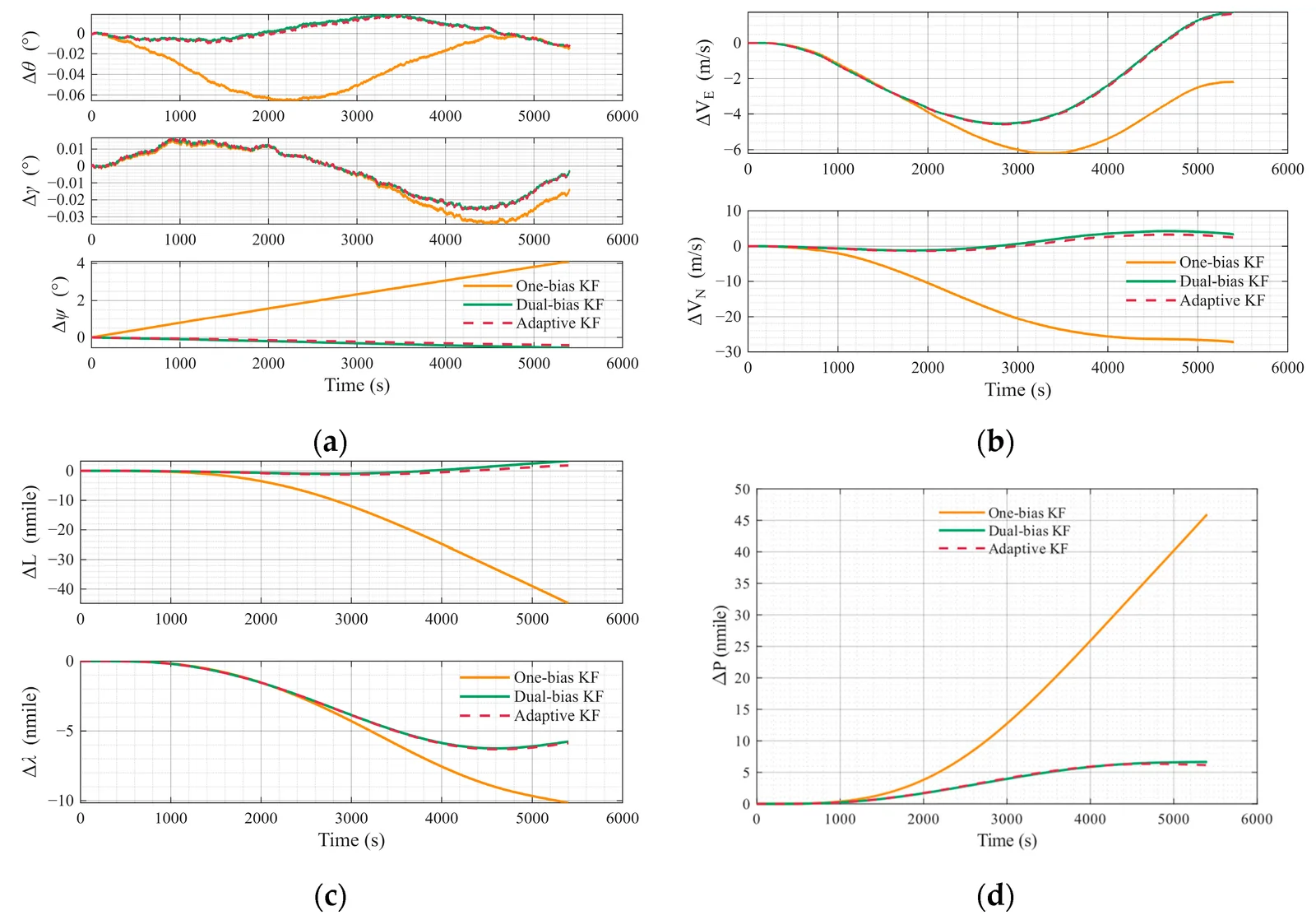
Static navigation results. (**a**) The attitude error of pitch, roll, and yaw from top to bottom. (**b**) The velocity errors of east and north are shown in the upper and bottom images, respectively. (**c**) The latitude error is shown in the first image, and the longitude error is shown in the second image. (**d**) The positioning error.

Based on the bias estimation results obtained in Section 4.2, the estimated gyroscope biases are compensated in the raw inertial measurements to form corrected observations, which are then processed by the navigation algorithm to compute attitude, velocity and position solutions. Similar to the simulation analysis, the navigation performance of the hybrid navigation system is evaluated in this section by comparing three filtering schemes under the same static measurement data. The corresponding attitude, velocity, and position errors are presented in Figure 14 and Table 6, Table 7 and Table 8. The static position reference used to compute the navigation errors in Figure 14 and Table 8 was provided by the GNSS.

As shown in Figure 14a and Table 6, the maximum pitch, roll and yaw errors are reduced by 71.7%, 25.3% and 86.5% with the dual-bias KF, and by 73.9%, 24.4% and 89.9% with the adaptive KF, respectively. In Figure 14b and Table 7, the maximum east and north velocity errors are reduced by 26.7% and 84.4% with the dual-bias KF, and by 26.2% and 87.9% with the adaptive KF, respectively. As shown in Figure 14c,d and Table 8, the positioning errors are reduced by more than 85% after the inertial sensor biases are eliminated. Moreover, the positioning error can be further reduced by approximately 0.7% with the adaptive KF. The navigation results are approximately 5 nautical miles after 1 h of navigation, and approximately 7 nautical miles after 1.5 h.

The static experimental results in terms of bias estimation, filtering stability and navigation accuracy are qualitatively consistent with the simulation analysis in Section 3. Specifically, compared with the one-bias KF, the positioning error is reduced by 96.9% and 97.0% in the simulation (Table 5) and by 85.5% and 86.2% in the experiment (Table 8) by the dual-bias KF and the adaptive KF, respectively, verifying the navigation performance improvement. The compensation effect on the drift error is not perfect, mainly because the hybrid navigation system in this paper only calibrates the bias of one gyroscope using the single-axis CAIG, rather than implementing a comprehensive calibration of the full six-axis IMU including the accelerometers as a whole.

### 4.4. Discussion

Combined with the simulation and experimental results above, this section further discusses the mechanism underlying systematic error elimination and the practical constraints of the single-axis CAIG-aided inertial navigation scheme.

From the perspective of observability analysis, the one-bias framework assumes zero CAIG bias and takes only the bias of the conventional gyroscope as the state to be estimated. According to the observability criterion for linear systems, the observability matrix of a discrete system is defined as:

(19) $$O=\left[\begin{array}{c} H\\ H\Phi \end{array}\right]$$

The state transition matrix is an identity matrix Φ=I and the observation matrix is also an identity matrix H=I. The rank of the observability matrix is rank(O)=1, which is consistent with the state dimension, indicating full state observability. However, this theoretical conclusion is valid only under the assumption that the CAIG is drift-free and serves as an observation benchmark.

From the perspective of error propagation, the bias output ${\hat{\varepsilon }}_{IMUZ}$ of the one-bias estimation model satisfies ${\hat{\varepsilon }}_{IMUZ}=\varepsilon_{IMUZ}-\varepsilon_{CAIG}$. The coupling of the conventional gyroscope bias and the CAIG bias yields systematic deviation. If the CAIG itself exhibits time-varying drift and cannot maintain an absolute zero-reference state, this will give rise to error coupling and systematic calibration deviations. This introduces systematic deviation originating from the model structure, which cannot be eliminated by tuning filter parameters.

The dual-bias estimation model contains two states $\varepsilon_{{IMU}_{Z}}$ and $\varepsilon_{CAIG}$. A two-dimensional observation equation is established, where the observation matrix is $H=I_{2\times 2}$, and the observability matrix is:

(20) $$O=\left[\begin{array}{c} H\\ H\Phi \end{array}\right]=\left[\begin{array}{cc} 1 & 0\\ 0 & 1\\ 1 & 0\\ 0 & 1 \end{array}\right]$$

The rank of the observability matrix is rank(O)=2, which equals the dimension of the dual states. The model achieves decoupling of the conventional gyroscope bias and the CAIG bias, and eliminates the systematic deviation. Under simulation conditions, the convergence of the estimated CAIG bias toward its true value benefits from the complete prior knowledge that the true bias is predefined in the simulation. The filter yields stable estimation results because the initial state and covariance are properly constrained. The core purpose of introducing the CAIG bias into the state-space model is to break the error coupling originating from the one-bias estimation architecture and eliminate the structural calibration deviation.

**Figure 15:**
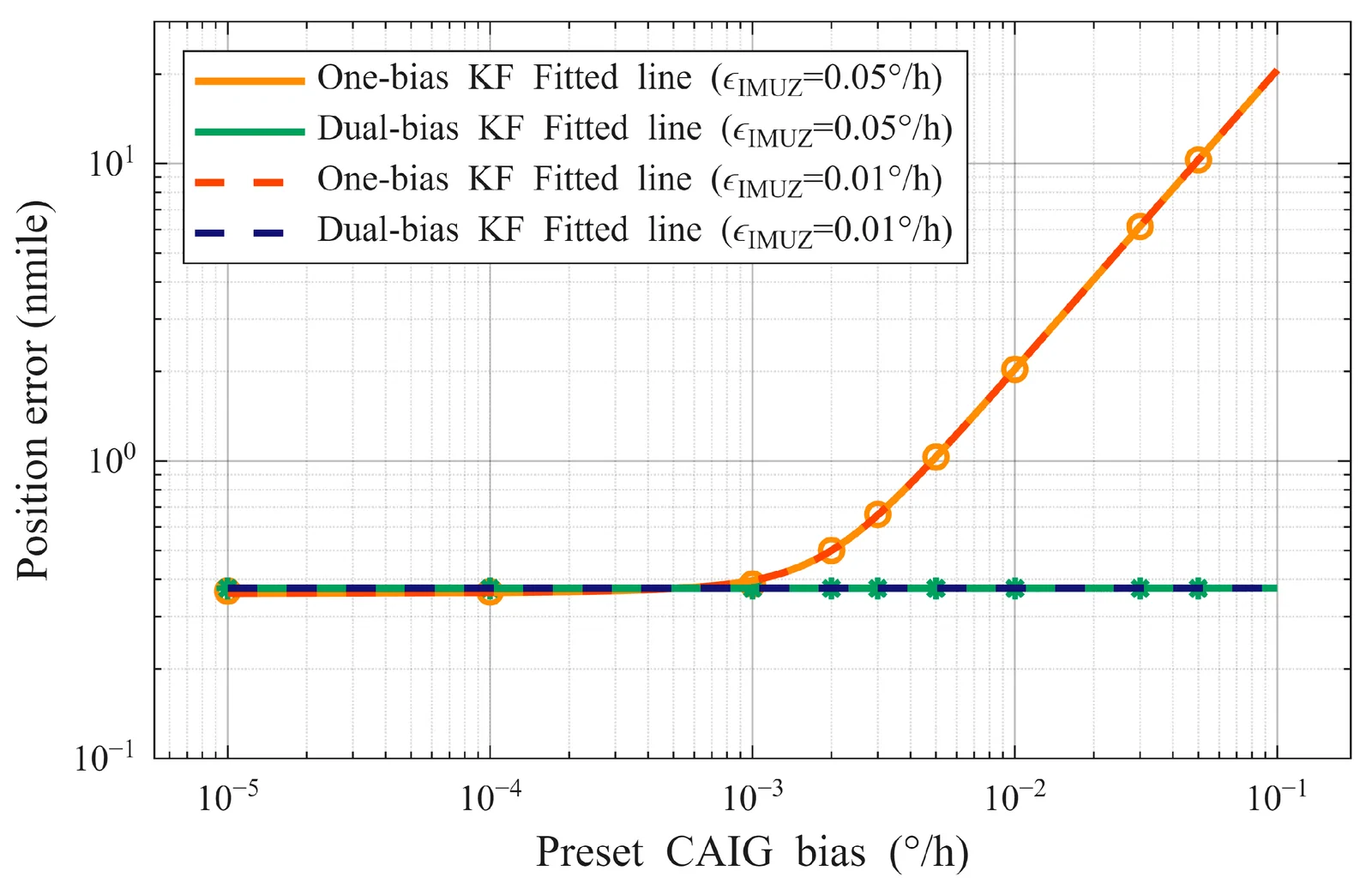
Position error versus the preset CAIG bias for the one-bias KF and the dual-bias KF under two conventional IMU bias levels. The dashed lines denote the results with $\varepsilon_{IMUZ}$ = 0.01°/h, and the solid lines denote those with $\varepsilon_{IMUZ}$ = 0.05°/h. The dual-bias curves remain nearly flat and coincide with each other regardless of the IMU bias level, whereas the one-bias curves rise rapidly once the CAIG bias exceeds approximately 0.001°/h.

To further quantify the applicable range of the proposed dual-bias estimation model, the preset CAIG bias is swept from 0.00005 to 0.05°/h under otherwise identical simulation conditions, and the resulting navigation position errors are compared in Figure 15 for two conventional IMU bias levels ($\varepsilon_{IMUZ}$ = 0.05°/h and 0.01°/h). When the CAIG bias is smaller than approximately 0.001°/h, the position error of the one-bias KF almost coincides with that of the dual-bias KF, indicating that the drift-free assumption on the CAIG is approximately valid in this regime and that the dual-bias model yields no appreciable additional benefit. As the CAIG bias increases beyond 0.001°/h, the one-bias KF suffers from severe error coupling, with the position error growing rapidly to the order of several nautical miles, regardless of the IMU bias level. In contrast, the dual-bias KF maintains a nearly constant low error level for both $\varepsilon_{IMUZ}$ values, and its two fitted curves almost overlap, confirming that the dual-bias joint modeling thoroughly decouples the conventional gyroscope bias from the CAIG bias. These results indicate that the structural advantage of the dual-bias estimation model mainly manifests when the CAIG bias exceeds approximately 0.001°/h.

## 5. Conclusions

A hybrid navigation system combining a CAIG with a conventional IMU is designed in this paper. Within this framework, the conventional IMU, comprising gyroscopes and accelerometers, serves as the primary sensor for the INS, while the CAIG acts as a high-precision sensor used to estimate the z-axis gyroscope bias of the conventional IMU. The dual-bias joint modeling incorporates both the conventional IMU gyroscope bias and the CAIG bias into the state vector, and effectively eliminates systematic deviations, thereby improving the navigation performance. Furthermore, the adaptive filtering algorithm enhances the accuracy and stability of the dual-bias estimation model. Finally, simulation and experimental results demonstrate that the designed single-axis hybrid navigation system leverages the precision of the CAIG and the high sampling rate of the conventional IMU. The single-axis hybrid navigation system reduces positioning errors by more than 85% after eliminating the conventional gyroscope bias, and achieves a navigation accuracy of approximately 5 nautical miles after 1 h of operation. The main limitation of this study is that the proposed model is validated under stationary conditions, while dynamic validation on a rotating platform or in a vehicle environment remains to be carried out. Future work will focus on improving the performance of the single-axis CAIG; meanwhile, a three-axis CAIG-IMU is under development, which features full-dimensional measurement capabilities for the angular rate and acceleration across orthogonal axes, to meet the requirements of quantum inertial navigation systems without external reference constraints.

## Figures and Tables

**Table 1:** Parameters of inertial sensors.

Inertial Sensor	Parameter
Conventional IMU bias	0.05°/h
Conventional IMU ARW	0.00005°/$\sqrt{h}$
Conventional IMU data output rate	100 Hz
CAIG bias	0.04°/h
CAIG ARW	0.00001°/$\sqrt{h}$
CAIG data output rate	100 Hz

**Table 2:** Bias estimation of the conventional IMU.

	Mean of Bias Estimation	Relative Error	STD of Bias Estimation
One-bias KF	0.0098°/h	80.4%	0.0172°/h
Dual-bias KF	0.0514°/h	2.8%	0.0170°/h
Adaptive KF	0.0513°/h	2.6%	0.0108°/h

**Table 3:** Simulated attitude error analysis of the navigation results.

	Maximum Error (°)			Reduction (%)		
	Pitch	Roll	Yaw	Pitch	Roll	Yaw
One-bias KF	0.00134	2.07	0.12	—	—	—
Dual-bias KF	0.00037	2.07	0.12	71.8%	0.1%	1.7%
Adaptive KF	0.00037	2.07	0.12	71.9%	0.1%	2.1%

**Table 4:** Simulated velocity error analysis of the navigation results.

	Maximum Error (m/s)		Reduction (%)	
	East	North	East	North
One-bias KF	0.74	1.67	—	—
Dual-bias KF	0.07	0.03	90.5%	98.2%
Adaptive KF	0.07	0.02	90.5%	98.4%

**Table 5:** Simulated position error analysis of the navigation results.

	Maximum Error (nmile)			Reduction (%)		
	Latitude	Longitude	Positioning	Latitude	Longitude	Positioning
One-bias KF	11.9	3.06	12.3	—	—	—
Dual-bias KF	0.02	0.38	0.38	99.8%	87.5%	96.9%
Adaptive KF	0.02	0.37	0.37	99.8%	87.7%	97.0%

**Table 6:** Attitude error analysis of the navigation results.

	Maximum Error (°)			Reduction (%)		
	Pitch	Roll	Yaw	Pitch	Roll	Yaw
One-bias KF	0.066	0.034	4.11	—	—	—
Dual-bias KF	0.019	0.026	0.56	71.7%	25.3%	86.5%
Adaptive KF	0.017	0.026	0.41	73.9%	24.4%	89.9%

**Table 7:** Velocity error analysis of the navigation results.

	Maximum Error (m/s)		Reduction (%)	
	East	North	East	North
One-bias KF	6.20	27.2	—	—
Dual-bias KF	4.55	4.24	26.7%	84.4%
Adaptive KF	4.58	3.31	26.2%	87.9%

**Table 8:** Position error analysis of the navigation results.

	Maximum Error (nmile)			Reduction (%)		
	Latitude	Longitude	Positioning	Latitude	Longitude	Positioning
One-bias KF	44.8	10.2	46.0	—	—	—
Dual-bias KF	3.32	6.25	6.65	92.6%	38.5%	85.5%
Adaptive KF	1.86	6.31	6.35	95.9%	37.9%	86.2%

## Data Availability

The datasets presented in this article are not readily available because they are owned by a third party and the terms of use prevent public distribution. Requests to access the datasets should be directed to guoqiang@cssc717.com.

## Abbreviations

The following abbreviations are used in this manuscript:

INS	Inertial Navigation System
IMU	Inertial Measurement Unit
CAIG	Cold Atom Interferometric Gyroscope
ARW	Angle Random Walk
KF	Kalman Filter
